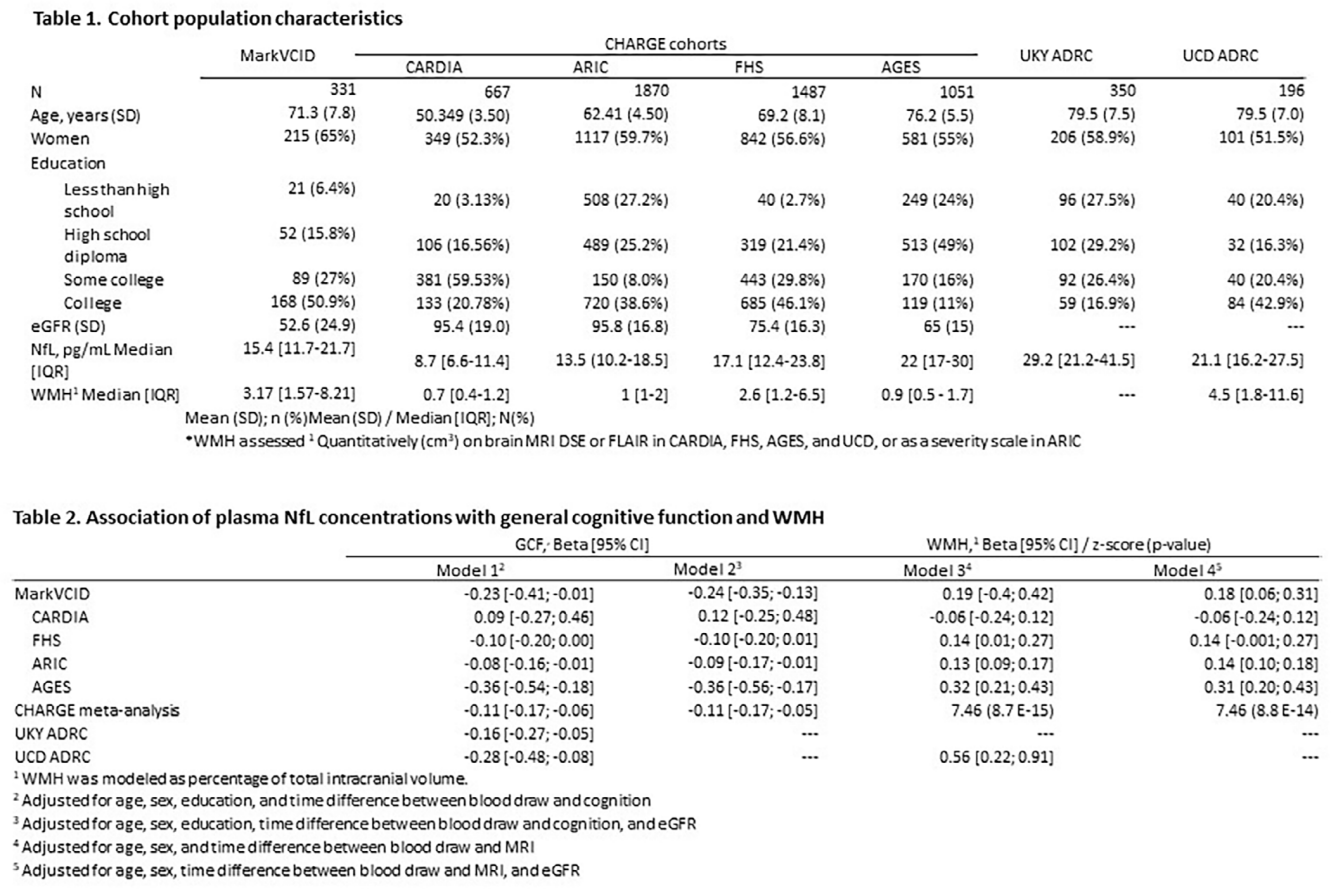# Plasma neurofilament light as a biomarker for vascular contributions to cognitive impairment and dementia

**DOI:** 10.1002/alz.086587

**Published:** 2025-01-09

**Authors:** Tiffany F. Kautz, Julia J Mathews, Rebecca Bernal, Chen‐Pin Wang, Qianqian Liu, Mitzi M. Gonzales, Russell P. Tracy, Danielle Parent, Donna M. Wilcock, Tiffany L. Sudduth, Danny JJ Wang, Abhay P Sagare, Gary A. Rosenberg, Hanzhang Lu, Joel H. Kramer, Charles Decarli, Lee‐Way Jin, Pauline Maillard, Herpreet Singh, Kristin Schwab, Karl HELMER, Steven M. Greenberg, Pia Kivisäkk, Hugo J. Aparicio, Alexa S Beiser, Saptaparni Ghosh, Myriam Fornage, Thomas H. Mosley, Djass Mbangdadji, Lenore J. Launer, Vilmundur Gudnason, Josh Bis, Bruce M. Psaty, Sudha Seshadri, Claudia L Satizabal

**Affiliations:** ^1^ Glenn Biggs Institute for Alzheimer’s & Neurodegenerative Diseases, University of Texas Health Science Center, San Antonio, TX USA; ^2^ Glenn Biggs Institute for Alzheimer's & Neurodegenerative Diseases, The University of Texas Health Science Center at San Antonio, San Antonio, TX USA; ^3^ Glenn Biggs Institute for Alzheimer's & Neurodegenerative Diseases, UT Health San Antonio, San Antonio, TX USA; ^4^ South Texas Veterans Health Care System, Geriatrics Research, Education & Clinical Center (GRECC), San Antonio, TX USA; ^5^ Glenn Biggs Institute for Alzheimer’s & Neurodegenerative Diseases, San Antonio, TX USA; ^6^ UT Health San Antonio, San Antonio, TX USA; ^7^ UT Health San Antonio, Glenn Biggs Institute, San Antonio, TX USA; ^8^ Cedars‐Sinai Medical Center, Los Angeles, CA USA; ^9^ University of Vermont, Colchester, VT USA; ^10^ University of Vermont, Burlington, VT USA; ^11^ Indiana University School of Medicine, Stark Neurosciences Research Institute, Department of Neurology, Indianapolis, IN USA; ^12^ University of Kentucky / Sanders‐Brown Center on Aging, Lexington, KY USA; ^13^ University of Southern California, Los Angeles, CA USA; ^14^ Zilkha Neurogenetic Institute, Keck School of Medicine, University of Southern California, Los Angeles, CA USA; ^15^ University of New Mexico, Albuquerque, NM USA; ^16^ Johns Hopkins University School of Medicine, Baltimore, MD USA; ^17^ Memory and Aging Center, UCSF Weill Institute for Neurosciences, University of California, San Francisco, San Francisco, CA USA; ^18^ University of California, Davis, CA USA; ^19^ University of California Davis Medical Center, Davis, CA USA; ^20^ Department of Neurology and Center for Neuroscience, University of California, Davis, CA USA; ^21^ Massachusetts General Hospital, Boston, MA USA; ^22^ Massachusetts General Hospital, Harvard Medical School, Boston, MA USA; ^23^ Boston University Chobanian & Avedisian School of Medicine, Boston, MA USA; ^24^ Boston University School of Medicine, Boston, MA USA; ^25^ University of Texas Health Science Center at Houston, Houston, TX USA; ^26^ University of Mississippi Medical Center, Jackson, MS USA; ^27^ National Institute on Aging, Baltimore, MD USA; ^28^ Icelandic Heart Association, Kopavogur Iceland; ^29^ University of Washington, Seattle, WA USA; ^30^ Department of Neurology, Boston University School of Medicine, Boston, MA USA; ^31^ Glenn Biggs Institute for Alzheimer's and Neurodegenerative Diseases and Department of Population Health Sciences, San Antonio, TX USA; ^32^ Glenn Biggs Institute for Alzheimer’s & Neurodegenerative Diseases, University of Texas Health Science Center at San Antonio, San Antonio, TX USA

## Abstract

**Background:**

The MarkVCID consortium was established to address the paucity of biomarkers for vascular contributions to cognitive impairment and dementia (VCID), a leading cause of dementia. Plasma neurofilament light (NfL), a neuroaxonal injury marker elevated in several neurological and neurodegenerative diseases, was selected as one of the first biomarkers to be examined. We performed comprehensive instrumental and clinical validation of the Quanterix Simoa NfL assay using the first MarkVCID cohort.

**Method:**

Plasma NfL was measured using HD‐X and HD‐1 Simoa instruments. Samples from the MarkVCID consortium were used to evaluate intra‐ and inter‐plate reliability, test‐retest repeatability, and inter‐site reproducibility. We used linear regression models to assess the association of NfL in MarkVCID with general cognitive function (GCF) as the primary outcome (n=331). In secondary analyses we assessed NfL associations with white matter hyperintensities (WMH). Models were adjusted for potential confounders, including eGFR as renal function influences NfL clearance. We replicated our findings using cohorts from the CHARGE consortium (CARDIA, ARIC, FHS, AGES; n=4,772), the UKY ADRC (n=350), and the UCD ADRC (n=196).

**Result:**

We found the Quanterix Simoa platform to be reliable with low coefficients of variation (average CV<12%), high inter‐site reproducibility (overall ICC = 0.93) and high repeatability in test‐retest samples drawn within 30 days (ICC=0.968). There was strong consistency across Quanterix instruments (HD‐X and HD‐1; R^2^≥0.98) and kits (N4PA and single molecule NfL; ICC≥0.81). We observed consistent significant associations between higher NfL concentrations and worse GCF in MarkVCID (β=‐0.23; [95% CI ‐0.41; ‐0.01), CHARGE cohorts (meta‐analysis β=‐0.11; [95% CI ‐0.17; ‐0.06]), the UKY ADRC (β=‐0.16; [95% CI ‐0.27; ‐0.05]) and the UCD ADRC (UCD: β=‐0.28; [95% CI ‐0.48; ‐0.08). Secondary analyses revealed significant associations between elevated NfL concentrations and higher WMH burden in MarkVCID (when controlled for eGFR), CHARGE, and the UCD ADRC.

**Conclusion:**

We have found that NfL can be reliably measured using the Quanterix platform, making this marker ideal for multi‐site clinical trials. We observed consistent associations for plasma NfL concentrations with cognition and WMH in MarkVCID and across independent samples, providing evidence that it can be a useful biomarker for stratification in VCID trials.